# Metastatic Supraclavicular Lymph Nodes among Patients with Lung Carcinoma in a Tertiary Care Centre: A Descriptive Cross-sectional Study

**DOI:** 10.31729/jnma.8188

**Published:** 2023-06-30

**Authors:** Suman Lamichhane, Ajit Thapa, Dinesh Chataut, Sundar Suwal, Mukhtar Alam Ansari, Birendra Kumar Yadav

**Affiliations:** 1 Department of Radiology and Imaging, Nepal APF Hospital, Balambu, Kathmandu, Nepal; 2 Department of Radiology and Imaging, Tribhuvan University Teaching Hospital, Maharajgunj, Kathmandu, Nepal; 3 Departmemt of Radiology and Imaging, National Medical College, Birgunj, Nepal; 4 Bir Hospital, National Academy for Medical Sciences, Kathmandu, Nepal

**Keywords:** *lung cancer*, *malignancy*, *ultrasonography*

## Abstract

**Introduction:**

Metastatic spread of lung cancer to supraclavicular lymph nodes is considered distant metastasis for treatment purposes. Detection of supraclavicular lymph node metastasis in patients with lung cancer serves for tissue diagnosis by itself and also helps avoid more invasive biopsy from the primary lung mass itself. Ultrasonography of the lower neck can detect supraclavicular lymph nodes before they are palpable and can also be used for safe sampling of these lymph nodes. The aim of this study was to find out the prevalence of metastatic supraclavicular lymph nodes among patients with lung carcinoma in a tertiary care centre.

**Methods:**

A descriptive cross-sectional study done in a tertiary care center, carried out from 15 September 2019 to 14 September 2020. Ethical approval was obtained from the Institutional Review Committee (Reference number: 84(611)E2/076/077). The study was done among 92 patients with biopsy-proven lung cancer (lung mass or supraclavicular lymph node biopsy) who were referred for evaluation, and/or percutaneous transthoracic biopsy. Convenience sampling method was used. Point estimate and 90% Confidence Interval were calculated.

**Results:**

Among 92 patients with proven lung cancer, metastatic supraclavicular lymph nodes were seen in 13 patients (14.13%) (8.17-20.09, 90% Confidence Interval). Among 13 patients with metastatic lymph nodes, 9 (69.23%) had palpable supraclavicular lymph nodes. The majority 11 (84.61%) had round-shaped lymph nodes. All metastatic lymph nodes showed loss of echogenic fatty hilum. A total of 12 (92.30%) metastatic lymph nodes showed a peripheral disorganized pattern of vascularity.

**Conclusions:**

The prevalence of metastatic supraclavicular lymph nodes was lower than in similar studies done in international settings.

## INTRODUCTION

Metastatic involvement of supraclavicular lymph nodes in a patient with lung carcinoma has an important implication for planning patient management because the involvement of these lymph nodes implies an unresectable disease.^1^ Lung cancer is the most common malignancy as well as the leading cause of cancer-related death in Nepal.^2,3^ Histological diagnosis of lung cancer is very important for optimal staging and therapy, sampling for which is often obtained under image guidance.^4^

Percutaneous lung biopsies have one of the highest complication rates of all imaging-guided biopsy procedures.^5,6^ Sampling from supraclavicular lymph nodes, if possible, can avoid such complications but still provide us with the histological diagnosis, as even small non-palpable supraclavicular lymph nodes can harbour metastases.^7^

The aim of this study was to find out the prevalence of metastatic supraclavicular lymph nodes among patients with lung carcinoma in a tertiary care centre.

## METHODS

This descriptive cross-sectional study was carried out in the Interventional Radiology suite of the Department of Radiology and Imaging, Tribhuvan University Teaching Hospital (TUTH) from 15 September 2019 to 14 September 2020. Ethical approval was obtained from the Institutional Review Committee (Reference number: 84(611 )E2/076/077). Consent was taken from each participant participating in the study. Patients who were suspected of having primary lung carcinoma based on clinical and/or radiologic findings and later proven by histopathological examination, who had no previous history of malignancy were included in this study. Patients with alternate diagnoses on histopathological examination or those patients who lost follow-up and whose histopathological examination reports were not available were not included in this study. Convenience sampling method was used. The sample size was calculated using the following formula:


$$\begin{array}{ll} \text{n} & ={\text{Z}}^{2}\times \frac{\text{p}\times \text{q}}{{\text{e}}^{\text{2}}}\\ & =1.64^{2}\times \frac{0.256\times 0.744}{0.10^{2}}\\ & =52 \end{array}$$

Where,

n = minimum required sample size
Z = 1.64 at 90% Confidence Interval (CI)
p = prevalence taken from previous study as, 25.56%^8^
q = 1-p
e = margin of error, 10%

The minimum required sample size was 52. However, the final sample size taken was 92.

After reviewing computed tomography (CT) images, all the patients were assessed with ultrasound sonography (USG) for supraclavicular lymph nodes. When supraclavicular lymph nodes were visible, sonographic morphology (shape, size, fatty hilum, vascularity) of the nodes was recorded. The visualised supraclavicular lymph nodes then underwent USG-guided biopsy or fine-needle aspiration biopsy (FNA). Biopsy from the lung lesion (CT or USG guided) was done in the patients without sonographically visible supraclavicular lymph nodes. The cytology or histopathology reports of the samples thus obtained were followed and recorded.

Data were collected in predesigned proforma and entered and analysed using IBM Statistics SPSS 23.0. Point estimate and 90% CI were calculated.

## RESULTS

Among 92 patients with proven lung cancer, metastatic supraclavicular lymph nodes were seen in 13 (14.13%) (8.17-20.09, 90% CI). Out of 13 metastatic lymph nodes, 9 (69.23%) were palpable and 4 (30.77%) were not palpable.

In histological examination majority of them showed metastatic carcinoma 3 (23.08%) (Table 1).

**Table 1:** Individual sampled lymph node report (n= 13).

Histology/Cytology of lymph nodes	n (%)
Metastatic adenocarcinoma with possible neuroendocrine differentiation (FNA)	1 (7.69)
Metastatic poorly differentiated carcinoma (FNA)	1 (7.69)
Metastatic carcinoma	3 (23.08)
Metastatic adenocarcinoma	2 (15.38)
Metastatic carcinoma, TTF-1^*^ positive suggesting adenocarcinoma of lung origin	1 (7.69)
Squamous cell carcinoma of lung origin	1 (7.69)
Metastatic squamous cell carcinoma	1 (7.69)
Metastatic tumour, clinical and radiological correlation advised	1 (7.69)
Moderately differentiated adenocarcinoma	1 (7.69)
Metastatic small cell carcinoma (IHC^†^ consistent with small cell carcinoma)	1 (7.69)

* Thyroid Transcription Factor-1

† Immunohistochemical study

Among 13 metastatic lymph nodes, 11 (84.61%) had round-shaped lymph nodes. All metastatic lymph nodes showed loss of echogenic fatty hilum 13 (100%). Mean short-axis diameter of the metastatic lymph nodes was 13.2±3.2 mm. Among them, 10 (76.92%) nodes showed short axis diameter ≥10 mm. Most of the metastatic lymph nodes 10 (76.92%) were round in shape. However, all the oval shaped metastatic lymph nodes showed short axis diameter ≥10 mm. All of the metastatic lymph nodes had absent fatty hilum. A total of 12 (92.30%) metastatic lymph nodes showed peripheral disorganized pattern of vascularity. No significant complications were seen during and immediately after the biopsy of the supraclavicular lymph nodes.

## DISCUSSION

In our study, the prevalence of metastatic supraclavicular lymph nodes was found to be 14.13%. The prevalence of metastatic supraclavicular lymph nodes in patients of lung cancer in our study was similar to a similar study which showed same prevalence to be 13.2%.^9^ However, the prevalence in our studies is lower as compared to many other studies.^8-12^

Detection of metastatic supraclavicular lymph nodes in lung cancer patients has an important implication in treatment planning as it would signify an unresectable disease (at least stage IIIb).^10^ Restaging of the lung cancer based on supraclavicular lymph nodal metastasis to a higher stage can help avoid surgeries which would add to patient morbidity only without any advantage of increasing survival. Moreover, tissue diagnosis can also be established by the biopsy of these metastatic lymph nodes for initiation of other therapy options available apart from surgery. Proven metastases in these nodes will also obviate the need for more complicated procedures undertaken for tissue diagnosis. Complication rates for biopsy or FNA of supraclavicular lymph nodes are notably low than that of percutaneous transthoracic lung biopsy.^8,10^ Routine USG evaluation of the lower neck focusing on the supraclavicular regions for enlarged lymph nodes can identify the non-palpable lymph nodes, which might have been missed on routine chest CT scans also. Ultrasound examination has been found in various studies to be more sensitive to palpation and CT scans for the detection of metastatic supraclavicular lymph nodes.^10^

The low prevalence of metastatic supraclavicular lymph nodes in our study could be due to the inclusion of the patients sent for percutaneous lung biopsy only. Supraclavicular lymph nodes are superficially located and are easily biopsied under ultrasound guidance with a very less complication rate as compared to transthoracic lung biopsy. We didn't find any complications in patients undergoing USG-guided biopsy of FNA of supraclavicular lymph nodes. No complications were observed in a similar study during the biopsy of supraclavicular nodes.^9^

Supraclavicular lymph nodes are superficially located and are easily biopsied under ultrasound guidance with a very less complication rate as compared to transthoracic lung biopsy. We didn't find any complications in patients undergoing USG-guided biopsy of FNA of supraclavicular lymph nodes. No complications were observed in a similar study during the biopsy of supraclavicular nodes.^8^

The limitation of the study was that the sample size was small. This study was conducted in a single centre, so the results may not be generalized to the whole population. Another limitation of this study is the inclusion of the lung cancer patients referred for percutaneous biopsy of lung lesion only, as supraclavicular lymph nodes were not assessed in those patients who underwent bronchoscopic biopsy for diagnosis of the lung cancer. We didn't record the complication rate of percutaneous lung biopsy in this study, so we couldn't find out the complication rate of supraclavicular lymph node FNA/ biopsy with the percutaneous lung FNA/biopsy.

## CONCLUSIONS

The prevalence of metastatic supraclavicular lymph nodes was lower than similar studies done in international settings. However, ultrasound examination of supraclavicular region can be an important assessment tool in evaluation of patients with suspected or proven lung cancer. In patients with radiologically and clinically suspected lung cancer, FNA or biopsy from supraclavicular lymph nodes is also useful for establishing the tissue diagnosis as well as staging before initiation of treatment in suspected lung cancer with less complication rate compared to the lung biopsy. Multicentre study with larger sample size and inclusion of all lung cancer patients regardless of the means of diagnosis is recommended.
